# Clinical usefulness of tools used for screening and measuring frailty in the ambulance and emergency department in older people: a scoping review protocol

**DOI:** 10.1136/bmjopen-2025-111454

**Published:** 2025-12-25

**Authors:** Carl Magnusson, Nellie Wahlstedt, Linda Östlundh, Anne Wissendorff Ekdahl, Karin Hugelius, Lisa Kurland

**Affiliations:** 1 Molecular and Clinical Medicine, Sahlgrenska Academy, University of Gothenburg, Gothenburg, Sweden; 2 School of Medical Sciences, Örebro University, Örebro, Sweden; 3 Örebro University Library, Örebro University, Örebro, Sweden; 4 Clinical Sciences, Faculty of Medicine, Lund University, Lund, Sweden; 5 Geriatric Department, Helsingborg Hospital, Helsingborg, Sweden; 6 Faculty of Medicine and Health, Örebro University, Örebro, Sweden

**Keywords:** Frailty, Emergency Departments, Frail Elderly

## Abstract

**Abstract:**

**Introduction:**

As people age, frailty becomes increasingly prevalent and is associated with adverse outcomes, including higher risks of hospitalisation, institutionalisation and mortality. Early identification of frailty in ambulance and emergency department (ED) settings may support clinical decision-making and help predict outcomes for older patients. However, there is currently no consensus on how frailty should be assessed in these settings, and the clinical usefulness of available tools remains uncertain. This protocol outlines the methods for a planned scoping review that aims to identify frailty screening tools used for older adults (age ≥65 years) in the ambulance and ED settings and to evaluate existing evidence on their clinical usefulness.

**Methods and analysis:**

This protocol follows the Preferred Reporting Items for Systematic Reviews and Meta-Analyses (PRISMA) extension for Protocols, and the final review will follow the Joanna Briggs Institute methodology and is reported in accordance with the PRISMA extension for Scoping Reviews guidelines. Studies will be included if they examine the use of frailty screening or assessment tools in individuals aged 65 years or older in ambulance and/or ED settings and report on aspects of clinical usefulness, such as feasibility, predictive validity or influence on clinical decisions. A comprehensive literature search will be conducted in PubMed, Embase, CINAHL, Scopus and Web of Science for studies published in English between January 2014 and December 2025. To refine the search strategy, an initial systematic pre-search was performed in PubMed using Medical Subject Headings terms, followed by a pilot study. A sample pilot screening of 101 references identified in the pre-search was conducted as a support for finalising the search. Ten of the papers in the pre-screening were furthermore used as a support for testing and validating the data extraction variables and quality assessment procedures. In the full scoping review, study selection and data extraction will be independently conducted by two reviewers using the Covidence software (Veritas Health Innovation, Melbourne, Australia), with any discrepancies resolved by a third reviewer. Extracted data will be summarised in tabular format and analysed through narrative synthesis. The methodological quality of included studies will be evaluated using the quality assessment tool of the National Heart, Lung, and Blood Institute for cohort studies.

**Ethics and dissemination:**

Ethical approval is not required as no primary data will be collected. Findings will be disseminated through publication in a peer-reviewed journal, conference presentations and summaries shared with relevant clinical and research-related stakeholders.

**STRENGTHS AND LIMITATIONS OF THIS STUDY**

- This scoping review is the first review to systematically examine the clinical usefulness of frailty assessment tools in ambulance and emergency department settings.
- The review follows the Joanna Briggs Institute methodology and the Preferred Reporting Items for Systematic Reviews and Meta-Analyses extension for Scoping Reviews reporting guidelines to ensure methodological rigour and transparency.
- A comprehensive search strategy will be applied across multiple major databases, increasing the likelihood of capturing a broad and relevant evidence base.
- Limiting inclusion to English-language publications may exclude relevant studies published in other languages.

## Introduction

 As people age, healthcare systems adapt to meet the needs of an increasing proportion of older adults globally, many of whom present with complex and progressively increasing care needs. By 2030, one in six people worldwide will be aged 60 years or older, and this proportion is expected to rise significantly by 2050.^1^ Almost every country, especially developing countries, is experiencing this demographic transition, contributing to a shift of healthcare demands. Older adults represent a substantial share of emergency department (ED) visits, and their care is often complicated by multimorbidity, functional decline and frailty.^2 3^

Frailty is a clinical state characterised by an increased risk of adverse outcomes such as dependency, institutionalisation and mortality when exposed to stressors, be they physiological, social or psychological.^4^ While frailty becomes more common with advancing age, it can occur across the entire adult lifespan.^5^ The syndrome arises from a progressive decline in reserve capacity in multiple physiological systems, resulting in reduced homeostatic reserves and increased risk of negative health events.^6^ As such, early identification of frailty is essential to tailor care, avoid interventions which do not benefit the older person, and support proactive care planning. In clinical settings, frailty assessment is increasingly recognised as a valuable tool to guide decision-making, prognostication and resource allocation.^7^ It offers the potential to personalise care by identifying patients who benefit from a holistic, goal-directed treatment approach and, conversely, from more intensive interventions despite high chronological age.^8 9^

In recent years, research on frailty assessment in emergency care has accelerated, with tools generally falling into three conceptual categories: phenotype-based models, cumulative deficit indices and clinician-judgement-based instruments. Among these, the Clinical Frailty Scale (CFS) and the Frailty Index are the most commonly used in ED settings.^10^ Several frailty screening tools such as the CFS, Deficit Accumulation Index, Identification of Seniors At Risk and the Study of Osteoporotic Fracture index have been evaluated in ED populations. However, evidence regarding their diagnostic accuracy and predictive value remains limited. A systematic review identified associations between frailty and adverse outcomes such as hospitalisation, institutionalisation, prolonged ED stays and mortality, but found no consistent link to short-term outcomes, for example, 30-day ED revisits.^11^ Despite growing awareness of the importance of frailty recognition in acute care, routine assessment remains uncommon in both ambulance services and EDs,^8 12^ and there is still no consensus on the most appropriate tools for these settings.^10^

The evidence for systematic frailty screening in emergency care has been questioned.^13^ More recently, a systematic review and meta-analysis demonstrated that Preferred Reporting Items for Systematic Reviews and Meta-Analyses (PRISMA)-7 and CFS were feasible in the ED and showed promising sensitivity and specificity when validated against comprehensive geriatric assessment.^14^ Since the publication of this review in 2022, a large body of literature has been published. The present scoping review aims to map and synthesise recent literature on frailty assessment in both ED and prehospital (ambulance) settings with respect to the clinical usefulness.

### Scoping review objective

Although previous reviews have mapped which tools exist, questions remain about their clinical usefulness, which is defined in the current review as accuracy, validity, reliability and feasibility. Therefore, this scoping review aims to address this gap by synthesising evidence on available instruments and their clinical usefulness in ambulance and ED care environments.

Specifically, we aim to identify screening tools for frailty used in the ambulance and ED for old people and to evaluate the evidence of their clinical usefulness.

## Methods and analysis

Given the heterogeneity of frailty assessment tools and the limited evidence regarding their clinical application in emergency settings, a scoping review is well suited to map the breadth of existing literature. This approach allows for the identification of knowledge gaps, variation in implementation and areas for future research. Scoping reviews are particularly useful when concepts are complex and evidence is emerging across diverse study designs to create an overview and map the existing knowledge.^15^ The proposed scoping review will be conducted in accordance with the JBI methodology for scoping reviews.^15^ The review will be reported in accordance with the 2020 PRISMA extension for Protocols and PRISMA extension for Scoping Reviews.^16^ The literature search is guided by the PRISMA extension for searches.^17^

### Inclusion/exclusion criteria

#### Inclusion

##### Participants

This review will consider studies that include older persons (≥65 years) cared for in the ambulance and ED setting, representing an unselected study population—that is, not focused on a specific medical condition.

##### Context

The context of this review is screening and measurement of frailty in the ambulance and ED. If the frailty assessment has been conducted in the ED, even though the study is in the inpatient setting, it will be included in this review. The study will be conducted without any geographical limitations.

##### Concept

This review will only consider studies focused on screening tools for frailty used in the ambulance and ED, and consider the clinical usefulness of these frailty assessment tools in terms of accuracy, validity, reliability or feasibility. These terms were chosen as proxies of clinical usefulness as there is no consensus definition of clinical usefulness for these emergency settings.

##### Types of studies

This scoping review will only consider peer-reviewed, published original studies. Both experimental and quasi-experimental study designs, including randomised controlled trials, non-randomised controlled trials, before and after studies and interrupted time-series studies, will be considered. In addition, analytical observational studies, including prospective and retrospective cohort studies, case–control studies and analytical cross-sectional studies, will be considered for inclusion.

### Exclusion

#### Participants

Studies with participants reflective of a specific medical condition will be excluded from this review. Studies with participants below 65 years of age will also be excluded, unless results for participants 65 years and above are presented separately.

#### Context

Studies that do not focus on frailty assessment will be excluded from this review. Studies not set in the ED or ambulance setting will also be excluded, as well as studies set in the psychiatric emergency setting.

#### Types of studies

Qualitative studies will not be included in this review, as the review seeks to assess the knowledge primarily concerning quantitative measures, as well as any type of review or meta-analysis. Grey materials, such as editorials, book chapters, conference proceedings, pre-prints and opinion papers, will also be excluded from this review.

### Information sources and search strategy

A search of PubMed,^18^ Open Science Framework,^19^ PROSPERO^20^ and medRxiv^21^ was conducted to ensure that no systematic review or scoping review on the topic was underway or was published. Initially, a systematic pre-search using PubMed and PubMed’s Medical Subject Headings (MeSH) terms was conducted by an experienced medical librarian (LÖ) at Örebro University Library in collaboration with the research team. This pre-search informed the development of a preliminary search string and selection of relevant search terms.

### Pilot

Following the initial pre-search, a pilot was conducted including the time period of January to March 2025 using the preliminary search string. This pilot aimed to validate the search strategy, data extraction variables and quality assessment tool prior to the full-scale search. The pilot involved a comprehensive and systematic literature search executed in March 2025 by the medical librarian (LÖ), with active collaboration from the research team. Three databases, PubMed (National Library of Medicine), Scopus (Elsevier) and Web of Science (Clarivate) were searched. The search combined MeSH terms and keywords from title and abstract fields, adapted specifically to the indexing system of each database. Full details of the pilot search strategies, including terms, filters and annotations, are provided (online supplemental table 1). The pilot search was restricted to studies published in English, between 1 January 2025 and 21 March 2025.

The pilot yielded 183 records, with 82 duplicates removed, leaving 101 studies for title and abstract screening. After screening, 10 studies progressed to full-text review and subsequent data extraction and quality assessment. Data extraction variables were predefined in an Excel file and subsequently uploaded into the blinded extraction module of Covidence (Veritas Health Innovation, Melbourne, Australia). Variables included general publication details (author, year, title, journal, country, sample size and study design) and specific data relevant to the research question (study aim, participant age, frailty tool used, assessment setting, type of assessment and clinical usefulness measures, including accuracy, validity, reliability, feasibility, outcomes and conclusions). The pilot confirmed the feasibility of the search strategy, quality assessment tool and highlighted minor adjustments necessary to refine the search terms further.

### Final search strategy

Based on the pilot results and subsequent discussions, minor refinements to MeSH terms and keywords were agreed on by consensus among the research group. A comprehensive final search will be conducted by the medical librarian (LÖ). Five databases, PubMed, Embase, CINAHL, Scopus and Web of Science, were systematically searched, using optimised combinations of MeSH/thesaurus terms and keywords searched in the title and abstract fields. The search will cover English language studies published from January 2014 to December 2025. Additionally, reference lists of included studies will be manually screened by two independent researchers (CM and KH) to identify any further relevant publications. The search strategy details, including exact terms, combinations, filters applied and annotations, will be transparently reported in the final review manuscript. An updated search will be conducted in all included sources prior to manuscript completion to ensure currency and comprehensiveness of citations.

### Source of evidence selection

All retrieved references from the final search will be collated and imported into Covidence systematic review software, where duplicates will be automatically identified and removed. Two independent reviewers (CM and KH) will perform title and abstract screening against the predefined inclusion criteria, followed by independent full-text review of selected citations. Reasons for exclusion at full-text review will be documented. Any disagreements will be resolved by a third, independent senior reviewer (LK). All stages of the selection process will be blinded to minimise bias. The results of the search and selection processes will be comprehensively reported in the final review and visualised using a PRISMA flow diagram.

### Data extraction

Predefined data extraction variables were defined and tested during the pilot stage, with minor adjustments implemented based on the pilot outcomes. Final extraction variables will be clearly defined by the research team and uploaded into Covidence prior to the main data extraction process. Data will include publication details and variables pertinent to the research question, validated through pilot testing. Two reviewers (CM and KH) will independently extract the data, with disagreements resolved by a third senior researcher (LK). Authors of original studies will be contacted for missing data when necessary.

### Data analysis and presentation

Extracted data concerning the study characteristics will be presented for each study in a tabular format to provide an overview of the different frailty instruments. Data extraction of accuracy, validity, reliability and feasibility will be presented in a numerical, tabular format for studies reporting these variables. Results will be presented in tabular format and narrative synthesis. The evidence of each tool’s use in the ambulance and the ED will be discussed and evaluated.

### Quality assessment of individual studies

To add to the value of the scoping review, we plan to include a quality assessment of the included studies. We plan to use the quality assessment tool of the National Heart, Lung, and Blood Institute (NHLBI) for observational cohort and cross-sectional studies.^22^ The NHLBI tool comprises 14 criteria evaluating key methodological domains such as study objectives, population clarity, recruitment procedures, exposure measurements, outcome assessments, confounding adjustment and overall methodological quality rating (good/fair/poor). Potential amendments made to the original assessment template (22) will be transparently documented and presented. In addition, depending on the findings of the literature search, we may need to adapt the quality assessment tool to accommodate other relevant study designs. The quality assessment of each study will be independently performed within Covidence by two blinded reviewers (CM and KH), and discrepancies will be resolved by a third senior reviewer (LK). The results of the assessment, including the final quality rating of each study, will be presented in an Appendix and summarised in the Results section.

## Ethics and dissemination

As this scoping review involves analysis of publicly available literature and does not include human participants or primary data collection, formal ethical approval is not required. The review will synthesise current evidence on the clinical usefulness of frailty assessment tools in ambulance and ED settings, with the aim of informing both clinical practice and future research. Findings will be submitted for publication in a peer-reviewed journal and disseminated through relevant professional and academic channels, including conferences, mailing lists and social media. Key results will also be summarised in accessible formats for stakeholders in emergency care and health policy. Any amendments to this protocol will be clearly documented, dated and explained in all resulting publications.

### Patient and public involvement

This work is a protocol of a planned scoping review of published literature. Patients and members of the public were not involved in its design. No patients will be recruited, and there was no patient involvement in defining the research questions, selecting outcomes or plans for dissemination at this stage.

## Supplementary material

10.1136/bmjopen-2025-111454 online supplemental file 1
