# Supplementary material for: Clinical usefulness of tools used for screening and measuring frailty in the ambulance and emergency department in older people: a scoping review protocol
Source: BMJ Open. 2025 Dec 25;15(12):e111454. doi: 10.1136/bmjopen-2025-111454 (PMC12742130; doi:10.1136/bmjopen-2025-111454)
Supplement: online supplemental file 1 [file bmjopen-15-12-s001.docx]

**Supplemental table 1**

**Search strategy for pilot screening:** Clinical usefulness of tools used for screening and measuring frailty in the ambulance and emergency department in older people - a scoping review protocol

**Source:** PubMed, Scopus and Web of Science

**Search specifications:** A filter for studies in English language only and a publication year filter for studies published in 2025 is applied in all databases.

| **Source and search date** | **Search string** | **Results and notes** |
| --- | --- | --- |
| PubMed  (NLM)  Search date: 2025-03-21 | ((("Emergency Medical Services"[Mesh] OR "Emergency Responders"[Mesh] OR "Emergency Medicine"[Mesh] OR "Evidence-Based Emergency Medicine"[Mesh] OR "Emergency Service, Hospital"[Mesh] OR "Ambulances"[Mesh] OR "emergency room*"[Title/Abstract] OR "emergency department*"[Title/Abstract] OR "emergency ward*"[Title/Abstract] OR "emergency patient*"[Title/Abstract] OR "ED"[Title/Abstract] OR "ER"[Title/Abstract] OR "emergency medicine"[Title/Abstract] OR "emergency medical service*"[Title/Abstract] OR "emergency unit*"[Title/Abstract] OR "emergency outpatient unit*"[Title/Abstract] OR paramedi*[Title/Abstract] OR "emergency responder*"[Title/Abstract] OR "emergency first responder*"[Title/Abstract] OR "acute care"[Title/Abstract] OR emergicenter*[Title/Abstract] OR "prehospital"[Title/Abstract] OR "emergency health service*"[Title/Abstract] OR "Emergency care"[Title/Abstract] OR "hospital emergency service*"[Title/Abstract] OR "emergency hospital service*"[Title/Abstract] OR ambulance*[Title/Abstract]) AND (frail*[Title/Abstract] OR fragil*[Title/Abstract] OR "Frailty"[Mesh] OR "Frail Elderly"[Mesh])) AND ("clinical deecision-making"[Title/Abstract] OR "medical decision-making"[Title/Abstract] OR "clinical decision making"[Title/Abstract] OR "medical decision making"[Title/Abstract] OR score*[Title/Abstract] OR scale*[Title/Abstract] OR index[Title/Abstract] OR indicator*[Title/Abstract] OR tool[Title/Abstract] OR tools[Title/Abstract] OR checklist*[Title/Abstract] OR screen*[Title/Abstract] OR assess*[Title/Abstract] OR measure*[Title/Abstract] OR diagnos*[Title/Abstract] OR determine*[Title/Abstract] OR "medical decision making"[Title/Abstract] OR "clinical decision making"[Title/Abstract] OR prevalence[Title/Abstract] OR prognosis[Title/Abstract] OR validity[Title/Abstract] OR accuracy[Title/Abstract] OR reliability[Title/Abstract] OR validation[Title/Abstract] OR specificity[Title/Abstract] OR specificity[Title/Abstract] OR predictive[Title/Abstract] OR "likelihood ratio*"[Title/Abstract] OR "LR"[Title/Abstract] OR "likelihood function*"[Title/Abstract] OR "likelihood estimate*"[Title/Abstract] OR "area under the curve*"[Title/Abstract] OR "area under curve*"[Title/Abstract] OR AUC[Title/Abstract] OR data[Title/Abstract] OR "Data Collection"[Mesh] OR "Prevalence"[Mesh] OR "Prognosis"[Mesh:NoExp] OR "Sensitivity and Specificity"[Mesh] OR "Predictive Value of Tests"[Mesh] OR "Likelihood Functions"[Mesh] OR "Area Under Curve"[Mesh] OR "Diagnosis"[Mesh:NoExp] OR "Clinical Decision-Making"[Mesh]OR "Geriatric Assessment"[Mesh])) AND ("Aged"[Mesh:NoExp] OR "aged"[Title/Abstract] OR elderly[Title/Abstract] OR older[Title/Abstract] OR old[Title/Abstract]) | **Results:** 69  **Notes:** All search terms are searched in a combination of titel, abstract and MeSH (when available). |
| Scopus (Elsevier)  Search date: 2025-03-21 | ( TITLE-ABS-KEY ("emergency room*" OR "emergency department*" OR "emergency ward*" OR "emergency patient*" OR "ED" OR "ER" OR "emergency medicine" OR "emergency medical service*" OR "emergency unit*" OR "emergency outpatient unit*" OR paramedi* OR "emergency responder*" OR "emergency first responder*" OR "acute care" OR emergicenter* OR "prehospital" OR "emergency health service*" OR "emergency care" OR "hospital emergency service*" OR "emergency hospital service*" OR ambulance ) AND TITLE-ABS-KEY (frail* OR fragil*) AND TITLE-ABS-KEY ( "clinical decision-making" OR "medical decision-making" OR "clinical decision making" OR "medical decision making" OR score* OR scale* OR index OR indicator* OR tool OR tools OR checklist* OR screen* OR assess* OR measure* OR diagnos* OR determine* OR "medical decision making" OR "clinical decision making" OR prevalence OR prognosis OR validity OR accuracy OR reliability OR validation OR specificity OR specificity OR predictive OR "likelihood ratio*" OR "LR" OR "likelihood function*" OR "likelihood estimate*" OR "area under the curve*" OR "area under curve*" OR AUC OR data) AND TITLE-ABS-KEY ( "aged" OR elderly OR older OR old ) ) AND PUBYEAR = 2025 AND ( LIMIT-TO ( LANGUAGE , "English" ) ) | **Results:** 74  **Notes:** All search terms are searched in a combination of title, abstract and keywords (here marked as “TITLE-ABS-KEY”). |
| Web of Science (Clarivate)  **Search date:** 2025-03-21 | ( TOPIC ("emergency room*" OR "emergency department*" OR "emergency ward*" OR "emergency patient*" OR "ED" OR "ER" OR "emergency medicine" OR "emergency medical service*" OR "emergency unit*" OR "emergency outpatient unit*" OR paramedi* OR "emergency responder*" OR "emergency first responder*" OR "acute care" OR emergicenter* OR "prehospital" OR "emergency health service*" OR "emergency care" OR "hospital emergency service*" OR "emergency hospital service*" OR ambulance) AND TOPIC (frail* OR fragil*) AND TOPIC ("clinical decision-making" OR "medical decision-Making" OR "Clinical Decision Making" OR "medical decision making" OR score* OR scale* OR index OR indicator* OR tool OR tools OR checklist* OR screen* OR assess* OR measure* OR diagnos* OR determine* OR "medical decision making" OR "clinical decision making" OR prevalence OR prognosis OR validity OR accuracy OR reliability OR validation OR specificity OR specificity OR predictive OR "likelihood ratio*" OR "LR" OR "likelihood function*" OR "likelihood estimate*" OR "area under the curve*" OR "area under curve*" OR AUC OR data ) AND TOPIC ("aged" OR elderly OR older OR old ) ) AND PUBYEAR = 2024 AND ( LIMIT-TO ( LANGUAGE , "English" ) ) | **Results:** 40  **Notes:** All search terms are searched in a combination of title, abstract and keywords (here marked as “TOPIC”). |
| **Total no. of references identified:** | | **183** |
| **Total no. of references identified after automatic de-duplication in Covidence** | | **101** |
