# Supplementary material for: Clinical usefulness of tools used for screening and measuring frailty in the ambulance and emergency department in older people: a scoping review protocol
Source: BMJ Open. 2025 Dec 25;15(12):e111454. doi: 10.1136/bmjopen-2025-111454 (PMC12742130; doi:10.1136/bmjopen-2025-111454)
Supplement: Supplementary file 2 [file bmjopen-2025-111454.reviewer_comments.pdf]

PEER REVIEW HISTORY

BMJ Open publishes all reviews undertaken for accepted manuscripts. Reviewers are asked to complete a checklist review form and are provided with free text boxes to elaborate on their assessment. These free text comments are reproduced below.

ARTICLE DETAILS

Title (Provisional)

Clinical usefulness of tools used for screening and measuring frailty in the ambulance and emergency department in older people - a scoping review protocol

Authors

Magnusson, Carl; Wahlstedt, Nellie; Östlundh, Linda; Ekdahl, Anne Wissendorff; Hugelius, Karin; Kurland, Lisa

---

VERSION 1 - REVIEW

**Reviewer**	**1**
**Name**	**Atashzadeh-Shoorideh, Foroozan**
**Affiliation**	**Shahid Beheshti University of Medical Sciences School of Nursing and Midwifery, Nursing Management**
**Date**	**08-Nov-2025**
**COI**	**None**

This manuscript presents a well-conceived and methodologically robust scoping review protocol addressing the clinical usefulness of frailty assessment tools in ambulance and emergency department settings for older adults. The topic is highly relevant to acute and geriatric care and aligns well with BMJ Open's focus on transparency, reproducibility, and methodological rigor. The protocol adheres closely to PRISMA-P and JBI guidelines, with clear articulation of the planned search, screening, and synthesis procedures. Minor revisions are recommended to strengthen conceptual clarity around "clinical usefulness," refine inclusion criteria (particularly regarding qualitative evidence), and ensure transparency in quality assessment and synthesis methods. Overall, the protocol is well designed, feasible, and suitable for publication following minor revisions.

VERSION 1 - AUTHOR RESPONSE

- Minor revisions are recommended to strengthen conceptual clarity around "clinical usefulness,"

Thank you. We have clarified the concept of clinical usefulness in the

manuscript. Specifically, we added an explanation that accuracy, validity, reliability, and feasibility are used as proxies for clinical usefulness due to the lack of a consensus definition in emergency care settings.

- Refine inclusion criteria (particularly regarding qualitative evidence)

Thank you for this comment. We have clarified the inclusion criteria regarding qualitative evidence. Qualitative studies will not be included in this review, as the aim is to synthesise knowledge primarily concerning quantitative measures.

- Transparency in quality assessment and synthesis methods

Thank you. We have strengthened the transparency of our data synthesis and quality assessment procedures. Specifically, we added a new section titled "Data analysis and presentation," which outlines how extracted data will be organised, tabulated and synthesised. We also clarified the quality assessment process by adding to the section on quality assessment of individual studies.
